# Brain Banks Spur New Frontiers in Neuropsychiatric Research and Strategies for Analysis and Validation

**DOI:** 10.1016/j.gpb.2019.02.002

**Published:** 2019-12-05

**Authors:** Le Wang, Yan Xia, Yu Chen, Rujia Dai, Wenying Qiu, Qingtuan Meng, Liz Kuney, Chao Chen

**Affiliations:** 1 Center for Medical Genetics & Hunan Key Laboratory of Medical Genetics, School of Life Sciences, Central South University, Changsha 410078, China; 2 Child Health Institute of New Jersey, Department of Neuroscience, Rutgers Robert Wood Johnson Medical School, New Brunswick, NJ 08901, USA; 3 Psychiatry Department, SUNY Upstate Medical University, Syracuse, NY 13210, USA; 4 Chinese Academy of Medical Sciences and Peking Union Medical College, Beijing 100101, China; 5 Affiliated Hospital of Guilin Medical University, Guilin 541000, China; 6 National Clinical Research Centre for Geriatric Disorders, Xiangya Hospital, Central South University, Changsha 410000, China

**Keywords:** Neuropsychiatric disorders, Brain bank, Postmortem brain, Expression quantitative trait loci, GWAS interpretation

## Abstract

**Neuropsychiatric disorders** affect hundreds of millions of patients and families worldwide. To decode the molecular framework of these diseases, many studies use human **postmortem brain** samples. These studies reveal brain-specific genetic and epigenetic patterns via high-throughput sequencing technologies. Identifying best practices for the collection of postmortem brain samples, analyzing such large amounts of sequencing data, and interpreting these results are critical to advance neuropsychiatry. We provide an overview of human brain banks worldwide, including progress in China, highlighting some well-known projects using human postmortem brain samples to understand molecular regulation in both normal brains and those with neuropsychiatric disorders. Finally, we discuss future research strategies, as well as state-of-the-art statistical and experimental methods that are drawn upon **brain bank** resources to improve our understanding of the agents of neuropsychiatric disorders.

## Introduction

Neuropsychiatric and neurological disorders, such as schizophrenia (SCZ), bipolar disorder (BIP), major depression (MD), and Alzheimer's disease (AD), are the leading cause of disability worldwide [1]. However, for more than half a century, a stagnant understanding of their pathophysiology has blocked the development of effective and well-validated neuropsychiatric therapies. Yet, the characteristically high heritability of these disorders should inform us that an earnest understanding of the genetic mechanisms behind these diseases is essential [2], [3]. Genome-wide association studies (GWAS) are achieving huge successes in identifying disease-associated variants. For example, the Psychiatric Genomics Consortium (PGC; http://www.med.unc.edu/pgc) has identified hundreds of loci associated with SCZ [4], as well as dozens of loci associated with BIP [5] and MD [6], [7].

Although many disease-associated variants have been identified, most have small effect sizes and are located in non-coding regions, which hinders interpretation of their functions and disease implications. Quantitative trait loci (QTL) analysis integrates population-based human variation with genome-wide molecular information, such as gene expression [8], DNA methylation [9], histone modifications [10], or chromatin states [11]. QTL is a possible solution for deciphering the function of non-coding variants [12]. Interestingly, most QTL signals show strong tissue specificity [13]. For example, the non-coding variant *rs199347,* associated with Parkinson’s disease exclusively, affects the expression of protein-coding gene GPNMB (*Glycoprotein Nmb*) in the human brain while sparing other tissues [14]. Robust brain bank collections can facilitate the comprehensive molecular profiling needed to advance research in neuropsychiatric disorders.

Many prominent brain projects on neuropsychiatric disorders generated big data at multiple regulatory levels, including epigenetic markers and gene expression. Although these multidimensional data identified numerous functional genomic elements, challenges remain that impede our full understanding of the underlying molecular etiologies of neuropsychiatric disorders and limit our ability to translate this understanding into improving human health. Although brain tissue samples have become a critically valuable resource for neuropsychiatric studies, to our knowledge, there are only a few comprehensive reports on brain bank resources. Therefore, in this review, we present a summary of the most representative brain banks and brain projects, emphasizing how harnessing these new resources and technologies can refine our insight into the underlying mechanisms of neuropsychiatric disorders. For example, we will discuss brain expression quantitative trait loci (eQTL) analysis as a methodology to interpret the potential functions of GWAS signals identified in various brain disorders. We also discuss the insights and limitations of current brain studies. Finally, we propose best practices for analyzing postmortem brain samples to more accurately interpret the resulting multidimensional data, thereby augmenting future investigations.

## Brain banks

A brain bank is a centralized resource that collects and stores postmortem brain tissues. Brain banks share samples and clinical information with qualified researchers worldwide to advance brain studies in both basic research and clinical trials. Currently, hundreds of human brain banks worldwide are dedicated to the collection of human post-autopsy brain tissues [15]. These have been helpful in demystifying brain-related diseases, such as AD, SCZ, BIP, and MD. Although brain tissue collection is the cornerstone for brain studies, obtaining high-quality brain tissues can be problematic. To counter this and enable better access, large networks such as the Australian Brain Bank Network, BrainNet Europe [16], NeuroBioBank [17], and the UK Brain Banks Network, share technologies and brain sample information. These brain banks have collectively standardized disease diagnosis and tissue collection procedures [18]. Here, we introduce procedures for obtaining high-quality postmortem brain tissue followed by a brief overview of brain banks worldwide and in China.

### Working with high-quality postmortem brain tissues

Various factors critically impact the quality of postmortem brain samples [19]. For example, an extended time interval between death and acquisition, the postmortem interval (PMI), can lead to RNA degradation [20]. Effective and rapid brain tissue acquisition and long-term preservation requires precise and unified manipulation using anatomical, cryopreservation, and slicing technologies. Rapid autopsy programs based on round-the-clock autopsy greatly shorten the PMI. Many important parameters are used to determine brain tissue quality, including brain pH, as well as the integrity of DNA, RNA, and proteins [19]. In a strict autopsy environment, which often prolongs the process of sample acquisition, brain pH can notably affect the integrity of RNA and DNA [19]. While formalin-fixed samples tender brain DNA relatively efficiently, the yields of high-quality RNA is somewhat problematic. It is clear that acquiring and preserving high-quality postmortem brain tissues requires great skill and adherence to standard procedures.

Accurately segmenting brain regions is critical, since biological functions vary by brain regions. There are several brain regions highly related to neuropsychiatric cognitive and emotional dysfunction. For example, the dorsolateral prefrontal cortex (DLPFC) and the hippocampus manage cognitive processes including working memory, planning, and cognitive flexibility. The striatum can receive glutamatergic and dopaminergic inputs from multiple sources functional, in the cognitive and reward systems. Accurate definitions for landmarks and label boundaries are important based on our assumption of the close correspondence of brain function to anatomy. The human cerebral cortex is difficult to label due to the great anatomical variations in the cortical folds and the difficulties in establishing consistent and accurate reference landmarks across the brain. Brain banks classify brain regions according to the Brodmann atlas, which defines 52 cerebral cortex regions [21]. Although there are no clear ‘gold standards’ for measuring the accuracy of anatomical assignments, it is common to measure consistency across trained human observers and variability across co-registered landmarks.

### Brain banks worldwide

Although the study of human brains is as old as medicine, brain banks benefitting neuropsychiatric research today arise from international collaboration, guided by modern principles of ethics, quality, and safety with valid scientific aims. One of the most famous brain banks is the Netherlands Brain Bank (NBB) in Amsterdam (https://www.brainbank.nl/) [16]. The NBB was established in 1985 to collect human brain tissues from donors with various neurological and psychiatric disorders and also non-diseased donors. NBB had collected brain samples from more than 4000 donors. Launched in 2001, the BrainNet Europe consortium (https://www.neuropathologie.med.uni-muenchen.de/funktionen/bne/index.html) has 19 members from across the continent. The brain tissues and the corresponding anonymized summary of each donor's medical records support extensive national and international research projects. North America with a wealth of brain banking resources has over 50 brain banks including the Allen Institute for Brain Science (https://alleninstitute.org/), Harvard Brain Tissue Resource Center (https://hbtrc.mclean.harvard.edu/), and the Stanley Medical Research Institute (http://www.stanleyresearch.org/). Representative brain banks also include the New South Wales Tissue Resource Centre (Australia, https://nswbrainbank.org.au/about/nswbtrc), Tokyo Metropolitan Institute of Gerontology (Japan, http://www.tmig.or.jp/), and the Brain Bank of the Brazilian Aging Brain Study (Brazil, http://www2.fm.usp.br/gerolab_en/index.php).

### Brain banks in China

In China, the number of brain samples is quite limited. The creation of Chinese brain banks has recently become a priority for researchers. China’s Han population represents the world’s largest ethnicity and roughly 80% of East Asia’s population; yet brain data from this population is currently understudied and will prove a valuable resource within the global survey. However, brain banking in China is slowly developing, with the China Human Brain Banking Consortium established in 2014 at the International Workshop on Human Brain Banking in China [22]. So far, there are nearly one thousand brain samples from dozens of consortium members, including the Xiangya School of Medicine Brain Bank, the Zhejiang University of China Brain Bank, the Chinese Academy of Medical Sciences & Peking Union Medical College Human Brain Bank, and others. The consortium organizes conferences and workshops annually to build up a unified process for brain tissue acquisition and storage, discussing policy for sample sharing, and exchanging experiences and new findings [23].

Evolutionary perspectives can help us better understand the relationship between brain development and disease. Therefore, nonhuman primate (NHP) brain resources play an important role in distinguishing human brain-specific regions. The Nonhuman Primate Reference Transcriptome Resource (http://nhprtr.org/index.html) began in 2010 [24]. Its goal is to establish an NHP reference transcriptome consisting of transcriptome sequencing data from multiple nonhuman species, including *Papio anubis*, *Pan troglodytes*, *Macaca fasicularis*, *Gorilla gorilla*, and 11 other non-human primates. Within their protocol, 22 tissue types are collected from four brain regions (*i.e.*, cerebellum, frontal cortex, hippocampus, and temporal lobe). By comparing brain regions of humans to those of non-human primates, Doan *et al.* was able to identify human-specific social and behavioral traits associated with autistic spectrum disorder (ASD) that are regulated by the human accelerated genomic regions [25].

## Brain projects

**Figure 1:**
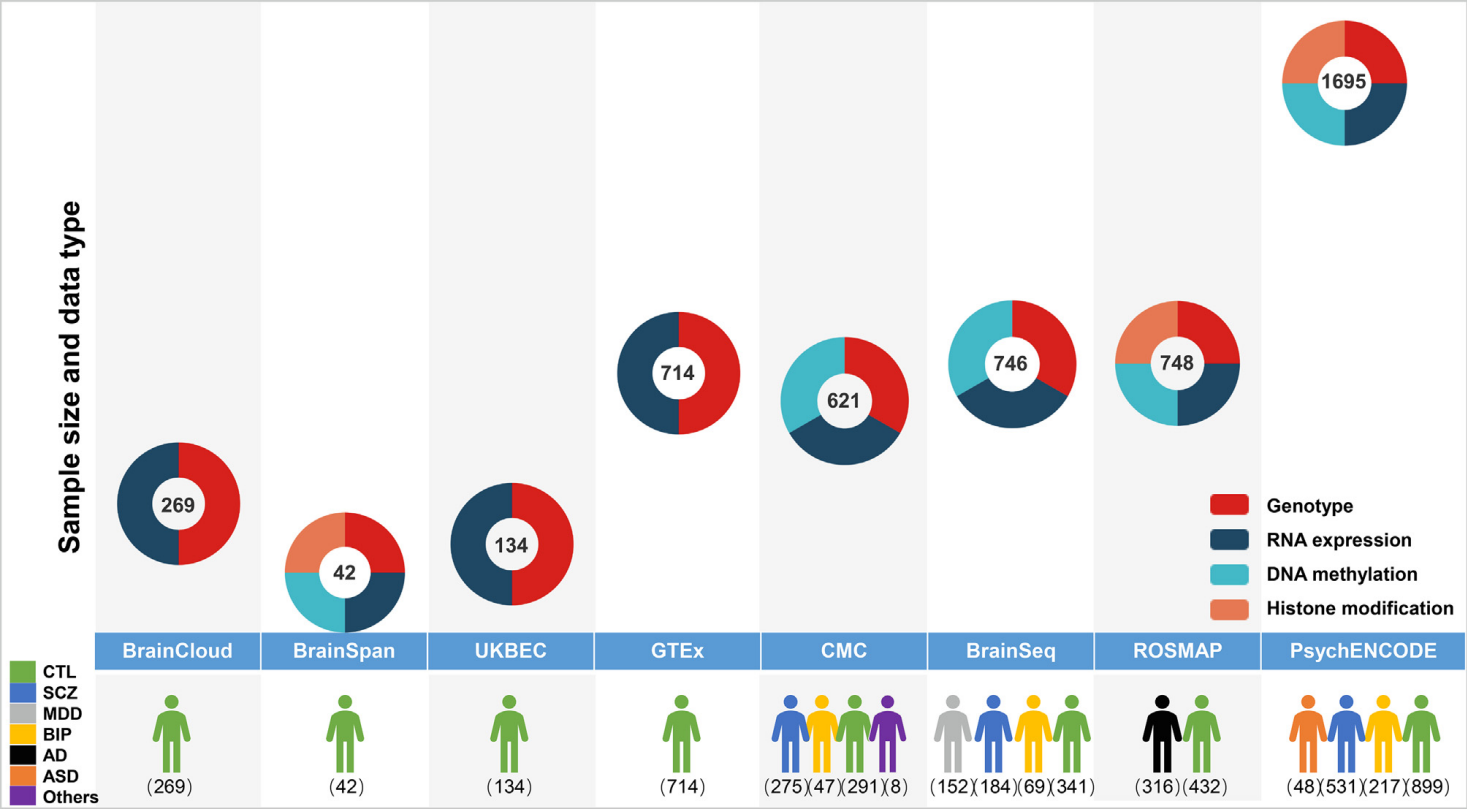
**Overview of the representative brain projects** Numbers in cycles indicate the number of brain samples used in each project. Different data types are indicated using different colors, which include genotype, RNA expression, DNA methylation, and histone modification data. Colors in the bottom panel indicate the distribution of healthy controls or patients with different diseases included in the respective projects. The projects and their web links for access were listed below. BrainCloud (http://braincloud.jhmi.edu/) [35]; BrainSpan (http://www.brainspan.org/) [27], [28]; UKBEC, UK Brain Expression Consortium (www.braineac.org/) [29]; GTEx, Genotype Tissue Expression Project (https://gtexportal.org/) [30]; CMC, CommonMind Consortium (commonmind.org/) [31]; BrainSeq (http://eqtl.brainseq.org/) [32]; ROSMAP, the Religious Orders Study and Memory and Aging Project (http://www.radc.rush.edu/) [33]. Only Capstone 1 data from PsychENCODE (http://www.psychencode.org/) were summarized in this figure. PsychENCODE Capstone 1 data comprise BrainGVEX, BrainSpan, CommonMind, UCLA- ASD, Yale- ASD, BipSeq, LIBD szControl, and CMC_HBCC datasets, but does not include fetal brain samples and outliers. CTL, control; SCZ, schizophrenia; MDD, major depressive disorder; BIP, bipolar disorder; AD, Alzheimer’s disease; ASD, autism spectrum disorder.

The collective increase in brain banks globally has spurred a multitude of brain research projects. For most projects, samples are obtained from well-constructed brain banks [26]. Brain research projects focus on many different dimensions, including brain development, spatiotemporal gene expression, epigenetic modification, and pathological characterization of neuropsychiatric disorders. Some of these efforts include, BrainSpan (http://www.brainspan.org/) [27], [28], UK Brain Expression Consortium (UKBEC, www.braineac.org/) [29], Genotype Tissue Expression Project (GTEx, https://gtexportal.org/) [30], CommonMind Consortium (CMC, commonmind.org/) [31], BrainSeq (http://eqtl.brainseq.org/) [32], the Religious Orders Study and Memory and Aging Project (ROSMAP, http://www.radc.rush.edu/) [33], PsychENCODE (http://psychencode.org/) [34], and BrainCloud (http://braincloud.jhmi.edu/) [35]. They aim to gather genotypic data and data at other regulatory levels for the human brain, to reveal the genetic regulatory mechanisms of the human brain at different levels (Figure 1 and Table 1, Table 2, Table 3).

**Table 1:** **Number of individuals across developmental stages per brain project**

**Stage**	**Age**	**BrainCloud**	**BrainSpan**	**UKBEC**	**GTEx**	**CMC**	**BrainSeq**	**ROSMAP**	**PsychENCODE**
Fetal	∼0	38	19	0	0	0	56	0	0
Infancy and childhood	0–12	34	12	0	0	0	31	0	65
Adolescence	12–20	49	4	2	0	3	60	0	88
Young adulthood	20–40	53	5	18	107	60	179	0	302
Middle adulthood	40–60	73	2	49	357	163	320	0	606
Late adulthood	≥ 60	22	0	65	250	395	100	748	634

*Note*: Only Capstone 1 data from PsychENCODE were summarized in this table. PsychENCODE Capstone 1 data comprise BrainGVEX, BrainSpan, CommonMind, UCLA- ASD, Yale- ASD, BipSeq, LIBD szControl, and CMC_HBCC datasets, but does not include fetal brain samples and outliers. UKBEC, UK Brain Expression Consortium; GTEx, Genotype Tissue Expression Project; CMC, CommonMind Consortium; ROSMAP, the Religious Orders Study and Memory and Aging Project.

**Table 2:** **Number of individuals by race per brain project**

**Race**	**BrainCloud**	**BrainSpan**	**UKBEC**	**GTEx**	**CMC**	**BrainSeq**	**ROSMAP**	**PsychENCODE**
European	112	21	134	608	500	–	730	1272
African American	147	14	0	91	90	–	14	350
Hispanic	6	4	0	0	26	–	0	41
Asian	4	1	0	8	4	–	3	20
Others	0	2	0	7	1	–	1	5

*Note*: “–”, data not available.

**Table 3:** **Number of samples per brain region per brain project**

**Brain region**	**BrainCloud**	**BrainSpan**	**UKBEC**	**GTEx**	**CMC**	**BrainSeq**	**ROSMAP**	**PsychENCODE**
Prefrontal cortex	269	37	127	129	621	746	748	1695
Temporal cortex	0	39	119	0	0	0	0	134
Anterior cingulate cortex	0	37	0	121	0	0	0	0
Cerebellum	0	35	130	173	0	0	0	0
Hippocampus	0	37	122	0	0	270	0	0
Caudate	0	0	0	160	0	500	0	0
Amygdala	0	36	0	100	0	0	0	0
Hypothalamus	0	0	0	121	0	0	0	0
Nucleus accumbens	0	0	0	147	0	0	0	0
Putamen	0	0	129	124	0	0	0	0
Substantia nigra	0	0	101	88	0	0	0	0

*Note*: Samples from BrainSpan, UKBEC, GTEx, BrainSeq, and PsychENCODE datasets were collected from multiple brain regions per individual.

Benefitting from the continual production of data and strengthened by in-depth structured analyses, brain projects are valuable references revealing basic functions as well as molecular and cellular pathologies related to neuropsychiatric disorders. As a source of data, each brain project offers unique design features and advantages for specific research aims. For instance, the GTEx project, which collects samples from non-disease tissue sites, including but not limited to the brain, focuses on tissue specificity of gene expression, cross-tissue gene expression regulation, and genetic variations that contribute to complex diseases and quantitative traits in humans [30]. The UKBEC, which collects samples from across a wide-range of brain regions, up to 12 regions per donor, focuses on the regulation and alternative splicing of gene expression [29]. BrainCloud [35] and BrainSpan [27], [28] focus on spatiotemporal gene expression regulation during the development of the human brain from embryonic to adult stages. Although BrainCloud is superior in terms of sample size, BrainSpan includes more brain regions and types of sequencing data, such as miRNA expression.

Other brain projects include samples from donors with or without neuropsychiatric disorders, exploring the differences between brain features of patients and those of controls. The Religious Orders Study (ROS) [36] and the Memory and Aging Project (MAP) comprise the ROSMAP project [37], a longitudinal, clinical, and pathological cohort study of aging and dementia. The ROS component focuses on data from various conditions of dementia within a limited population, while the MAP project focuses on reduced cognitive and motor function and disease risk of those with AD within a more varied population. CMC and BrainSeq [31], [32] focus on neuropsychiatric disorders, including SCZ, BIP, ASD, and MD, by comparing diseased samples with controls. The BrainSeq project seeks to identify therapeutic drug targets for neuropsychiatric disorders by understanding the genetic and epigenetic regulations across the human lifespan. The PsychENCODE project [34] makes an extensive, "multidimensional" genetic and epigenetic dataset available to the public, derived from the tissue samples of postmortem healthy and diseased human brains. The project characterizes disease-associated regulatory and genetic features within pathological models, focusing initially on ASD, BIP, and SCZ [38], [39], [40]. Current data generated from the PsychENCODE project include: chromatin immunoprecipitation following next-generation sequencing (ChIP-seq), RNA-seq, whole-genome bisulfite sequencing (WGBS), miRNA sequencing (miRNA-seq), isoform sequencing (IsoSeq), assay for transposase accessible chromatin with high-throughput sequencing (ATAC-seq), enhanced reduced representation bisulfite sequencing (ERRBS), single nucleotide polymorphism (SNP) genotypes, array methylation, and reverse phase protein array (RPPA).

The major findings using postmortem samples from brain projects are summarized in Table S1. These data provide important insights into the contribution of genetic and epigenetic factors to mechanisms underlying neuropsychiatric disorders. Particularly, the BrainSeq Consortium performed RNA-seq on 495 postmortem brains with ages across the human lifespan, including 175 samples from SCZ patients and 320 controls [41]. Through integrative analyses, this consortium demonstrates that 48.1% SCZ GWAS risk variants are associated with expression of nearby genes, and 237 differentially expressed genes implicated in synaptic processes are regulated in early brain development. The earlier study on the epigenetic landscape of frontal cortex in patients with SCZ [42] shows that SCZ-associated CpGs strongly correlate with fetal development stage rather than the adult stage of the brain. These results reveal potential SCZ pathogenesis in gene expression and DNA methylation during brain development and maturation. Moreover, recent studies by the PsychENCODE project have identified cell composition and maturation leading to spatiotemporal transcriptomic variation patterns in human and macaque brains [43]. They also observe associations of neuropsychiatric diseases with epigenetic markers [38], QTLs [39], and isoform-level changes [44]. For example, they have identified several interesting targets, including *DGCR5* and *POU3F2*, which play essential roles in regulating SCZ-related genes at the network level [45], [46]. These postmortem studies provide important insights into the genetic architecture for robust and informative models of neuropsychiatric disorders, which will help in devising strategies for novel therapeutics interventions.

## Strategies and execution

**Figure 2:**
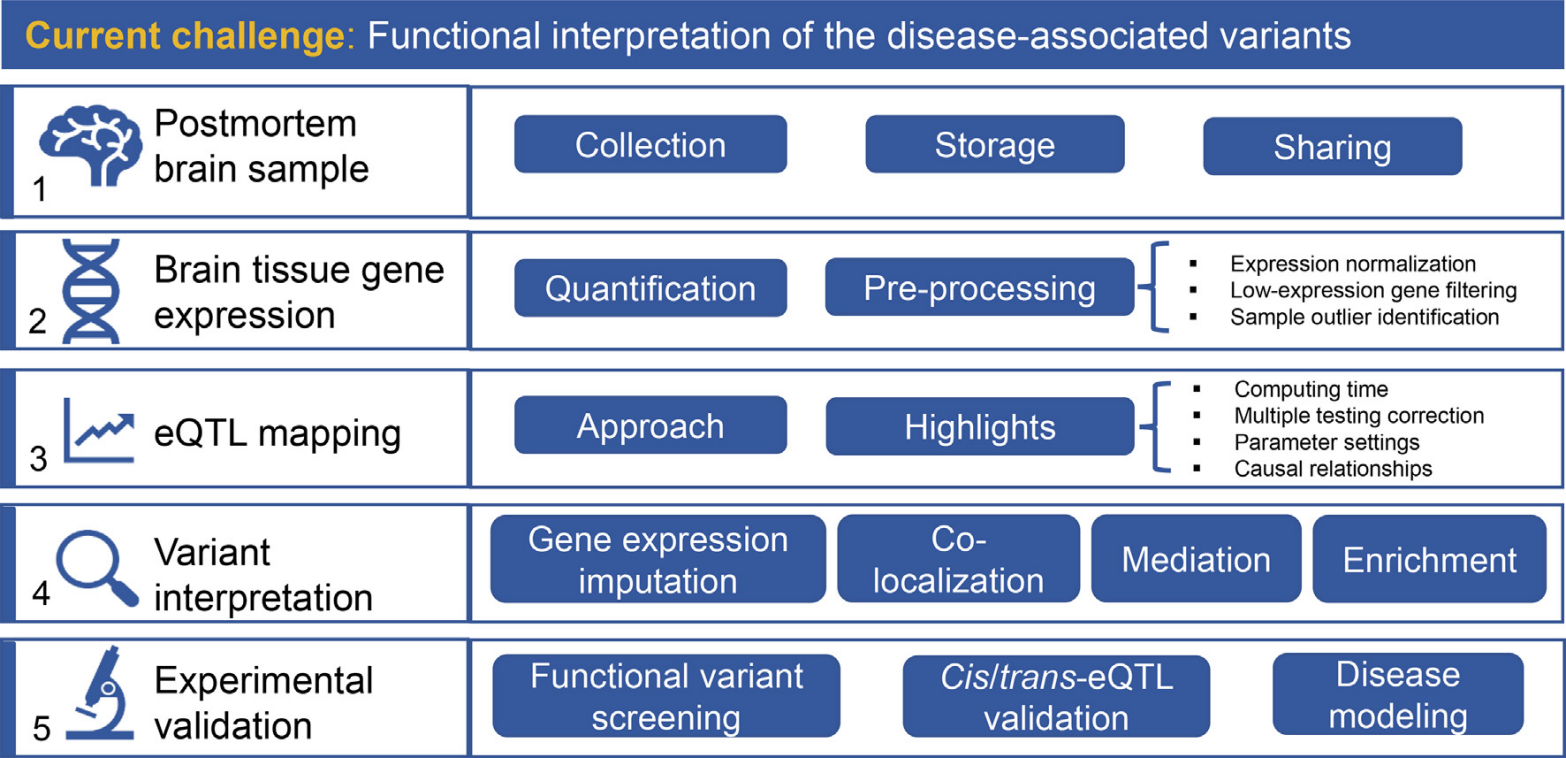
**Overview of strategies and methods in neuropsychiatric studies**

Unarguably, postmortem brain resources are valuable in revealing the biological underpinnings of neuropsychiatric disorders; however, unravelling the full potential of multidimensional brain data is still a great challenge. One promising strategy employs QTL analysis, which integrates population-based human variations with genome-wide molecular information (*e.g.*, gene expression, DNA methylation, histone modification, and chromatin states). Widely used, QTL captures the associations between genetic variants and gene expression. For instance, QTL can be used to investigate variants at *cis*-regulatory elements, such as transcription factor-binding regions, which confer differential expression of target genes. Combined with GWAS, QTL studies interpret how disease-associated variants may contribute to molecular traits and disease susceptibility. In this section, we will discuss eQTL specifically, summarizing the key steps for pre-processing of brain gene expression data, highlighting important issues in eQTL analysis, explaining how to use eQTL to interpret GWAS signals, and finally, introducing cutting-edge experiments to validate regulatory signals (Figure 2 Overflow of the research strategies and methods).

### Pre-processing brain gene expression data

Although laborious, data pre-processing is essentially the first step to ensure proper and efficient data modelling. A clean, software-compatible format will ensure reproducible results and save hours, even days, of data analysis [47]. Variable reporting of gene expression can arise from biological factors and technical variations. To distinguish biological variations from confounding factors, technical factors (*e.g.*, batch effects) must be removed or adjusted. Major pre-processing steps include gene expression normalization and filtering, sample outlier identification, and covariate correction. Because strategies in the human brain studies are the major focus of this article, we will only cover the key steps that may alter the quality of brain gene expression results. Comprehensive guidelines for gene expression data analysis are well discussed elsewhere [48], [49] and are beyond the scope of this review.

The first key step is gene quantification and filtering. Tools for quantification are widely available, such as Cufflinks [50], eXpress [51], Flux Capacitor [52], kallisto [53], RSEM [54], Sailfish [55], and Salmon [56]. Each tool can accurately assign reads to transcripts and quantify expression. These functions are vital for interpreting tissue-specific expression patterns in the brain [57]. However, the criteria for poorly expressed genes vary across studies. For instance, PsychENCODE project filters genes with transcript per million (TPM) < 0.1 in more than 25% of samples [58].

The second key step is sample outlier removal. Samples with a high degree of poorly expressed genes or gene expression patterns distinct from other samples are removed. This step can be carried out in dimension reduction analysis such as principal component analysis (PCA) and multidimensional scaling (MDS). Network concepts such as standardized connectivity (the overall strength of connections between a given sample and all of the other samples in a network) are also used to confirm sample outliers within a group [59].

The third key step is controlling covariates, including both known and unknown covariates. Known covariates can be either technical, such as batch effects, or biological, such as sex and age. Some biological covariates have been ignored by earlier research, leading to potentially confounding results. For instance, cell-type composition is one such common problem: since bulk-tissue RNA-seq only measures the average behavior, it is unable to capture cellular heterogeneity, which makes the observed changes in gene expression reflect only changes in cell-type composition, rather than fundamental changes in cell states [60]. Therefore, cell numbers and ratios of multiple cell types are important biological covariates, that affect brain gene expression profiles, since different cell states rather than cell type composition reflect distinct biological activities and gene expression patterns. Another covariate that is critical but often neglected is drug treatment history. Gene expression can vary dramatically across therapeutic courses. The unknown factors, also called hidden determinants, can reduce the power to find eQTLs. Surrogate variable analysis (SVA) [34] or probabilistic estimation of expression residuals (PEER) [61] can calculate unknown sources of variation, followed by a linear regression model to remove them. One could choose ComBat [62] (in R package *sva*) to remove the batch effects; finally, a linear regression model will remove the confounding factors.

### Pitfalls and promises in eQTL analysis

The aim of eQTL analysis or eQTL mapping is to characterize associations between the expression of corresponding genes and SNPs, thereby isolating specific regulatory regions within the genome. A variety of approaches have been proposed, including using linear regression, ANOVA, and non-linear models. Some approaches also account for pedigree and other confounding factors [63], integrating known functional elements [64], or considering allelic imbalances [65]. FastQTL, for instance, features expansive permutations that refine *P* values and reduce computational burden.

Several issues should be highlighted in eQTL analysis. The first is computing time. Pairwise association compares up to one million genetic variants to tens of thousands of genes, making analysis computationally intensive, especially when employing a non-linear model on a larger dataset. Secondly, multiple testing corrections become necessary for many of the tests performed. One common solution is to calculate the false discovery rate for each SNP-gene pair. Furthermore, separating the *cis*-eQTLs and *trans*-eQTLs is crucial, since local variants may regulate gene expression much more than distal variants. However, this correction alone is too strict because those tests are not biologically independent. Therefore, permutation-based methods, which create the null distribution of associations by tens of thousands of permutations, were developed to set up an effective threshold for identifying statistically significant eQTLs. Third, parameter settings can be a critical factor when comparing eQTLs across multiple studies. For example, the distance between SNPs and gene locations is used to differentiate *cis*-eQTL and *trans*-eQTL signals, which could be defined as 1 Mb, 5 Mb or 10 Mb in different studies. Varied distance settings may lead to different statistical burdens for SNPs located in regions ranging from 1 to 10 Mb and result in variable outcomes. The customized cut-off threshold for minor allele frequency (MAF) may also cause the loss of some true signals. Fourth, some eQTLs have such strong correlations with gene expression that they may not prompt gene expression changes. In other words, those genetic variants may be correlated with the causal variants due to linkage disequilibrium or other factors. Both statistical and experimental approaches have been proposed to solve this problem [66], [67]; either ways, it is critical to identify true causal variants when integrating eQTL and GWAS results [68].

### Interpreting GWAS signals

GWAS variants can increase or decrease gene expression, a culprit behind the etiology of many diseases; QTL helps us interpret how non-coding GWAS variants work. Several kinds of methods, each with unique principles, have been developed to integrate GWASs and eQTL results (Table 4). One type of method is based on gene expression imputation, such as PrediXcan [70] and transcriptome-wide association study (TWAS/FUSION) [71]. These methods estimate the genetically regulated component of expression using reference transcriptome datasets such as GTEx [30], GEUVADIS [8], and DGN [85] among others to build a database of prediction models. For each new genotype data, these methods impute gene expression and then correlate that gene expression to a trait of interest to identify trait-associated genes. The second group investigates the co-localization of GWAS causal variants and eQTL causal variants. For example, COLOC [72], MOLOC [73], ENLOC [86], HyPrColoc [74], and Sherlock [75] use a Bayesian statistical framework to integrate GWAS summary data and eQTLs to estimate the causal variants, and eCAVIAR [78] considers multiple causal variants within one locus. Other groups include enrichment methods, such as S-LDSC [82] and eQTLEnrich [81], and mediation methods. Summary data-based Mendelian Randomization (SMR) [66] and generalized SMR (GSMR) [84] test whether the effect of a GWAS SNP on a specific trait has been mediated by the expression of a gene.

**Table 4:** **Algorithms and software for integrating GWAS and eQTL data**

**Name**	**Description**	**LD considered**	**Programming language**	**Operatingsystem**	**Link**	**Ref.**
MetaXcan	Gene expression imputation	Y	Python	Unix/Linux	https://github.com/hakyimlab/MetaXcan	[69]
PrediXcan	Gene expression imputation	N	Python	Unix/Linux	https://github.com/hakyimlab/PrediXcan	[70]
TWAS / FUSION	Gene expression imputation	Y	R	Unix/Linux, Mac OS, Windows	http://gusevlab.org/projects/fusion/	[71]
COLOC	Co-localization	Y	R	Unix/Linux, Mac OS, Windows	https://github.com/chr1swallace/coloc	[72]
MOLOC	Co-localization	Y	R	Unix/Linux, Mac OS, Windows	https://github.com/clagiamba/moloc	[73]
ENLOC/fastENLOC	Co-localization	Y	Perl	Unix/Linux, Mac OS, Windows	https://github.com/xqwen/integrative	[73]
HyPrColoc	Co-localization	Y	R	Unix/Linux, Mac OS, Windows	https://github.com/jrs95/hyprcoloc	[74]
Sherlock	Co-localization	Y	-	Web interface	http://sherlock.ucsf.edu/	[75]
JEPEG	Joint eQTL analysis	Y	C++	Unix/Linux	https://dleelab.github.io/jepeg/	[76]
CAVIAR	Co-localization	Y	C	Unix/Linux, Mac OS, Windows	http://genetics.cs.ucla.edu/caviar/	[77]
eCAVIAR	Co-localization	Y	C	Unix/Linux, Mac OS, Windows	http://genetics.cs.ucla.edu/caviar/	[78]
GMAC	Mediation analysis	N	R	Unix/Linux, Mac OS, Windows	https://cran.r-project.org/web/packages/GMAC	[79]
FINEMAP	Co-localization	Y	C	Unix/Linux, Mac OS	http://www.christianbenner.com/	[80]
eQTLEnrich	Enrichment	Y	MATLAB	Unix/Linux, Mac OS, Windows	https://segrelab.meei.harvard.edu/software/	[81]
S-LDSC	Enrichment	Y	Python	Unix/Linux, Mac OS, Windows	https://github.com/bulik/ldsc	[82]
NEO	Structural equation model	N	R	Unix/Linux, Mac OS, Windows	https://labs.genetics.ucla.edu/horvath/htdocs/aten/NEO/	[83]
SMR	Mendelian randomization	Y	C	Unix/Linux, Mac OS, Windows	http://cnsgenomics.com/software/smr	[66]
GSMR	Mendelian randomization	Y	R	Unix/Linux, Mac OS, Windows	http://cnsgenomics.com/software/gsmr/	[84]

*Note*: eQTL, expression quantitative trait loci; TWAS, transcriptome-wide association study; JEPEG, joint effect on phenotype of eQTLs/functional SNPs associated with a gene; CAVIAR, causal variants identification in associated regions; eCAVIAR, eQTL and GWAS causal variants identification in associated regions; GMAC, Genomic Mediation Analysis with Adaptive Confounding Adjustment; NEO, Network Edge Orienting; SMR, summary-data-based Mendelian randomization; GSMR, generalized summary-data-based Mendelian randomization.

While using eQTL to interpret GWAS results is a good way to understand gene regulatory mechanisms, it is not without limitations. First, for some diseases if the most relevant tissue/cell types or developmental stages are not available in eQTL analysis, we can find neither the true genetic regulation nor the related genes. Second, gene expression is only one dimension of genetic regulation. If the biological mechanism is independent of gene expression levels but affects other regulatory cascades, such as splicing, chromosome accessibility, or ribosome profiling, eQTL alone will not be enough to explain the underlying processes. Third, QTL and GWAS focus on common variants, therefore they cannot capture rare variants with higher effect sizes in gene expression [87].

### Experimental approaches to characterize functional variants

After identifying disease risk variants or regulatory elements using the aforementioned bioinformatics analysis methods, the next step is to characterize the function of the variants.

To validate risk variants as the eQTL signal, using high-throughput and sensitive methods to measure their effect on gene expression is a widely adopted approach. As a favored method, reporter gene assay screening validates whether functional elements with eQTL signals regulate target gene expression, by cloning the regulatory elements into an expression reporter vector [74]. Whereas reporter assays validate regulatory functions of variance *in vitro*, CRISPR can be used to validate regulatory functions of the variance within native chromosome regions *in vivo*. For instance, Diao et al. used a CRISPR tiling-deletion-base genetic approach to identify some *cis*-regulatory elements in mammalian cells [88]. Furthermore, high throughput CRISPR screening systems, such as the CRISPR-Cas9, have been used to investigate the effect of the regulatory variance on the downstream target genes [75], [78], [81], [82], [84]. Recently, studies have refined the resolution of this technique, including the dCas9 fusion APOBEC1 (Apolipoprotein B mRNA Editing Enzyme Catalytic Subunit 1)/TadA (tRNA-specific adenosine deaminase)-mediated efficient single base mutation system [69], [87]. While CRISPR technology has these advanced capabilities, it is not without limitations. For instance, inconsistencies such as off-target genome editing (*i.e.*, inducing unwanted allelic variances) have been problematic to date [89]. Nonetheless, CRISPR has tremendous potential for single base screening and clinical applications. We are confident that CRISPR will mature into a dependable tool for correcting genetic variation in the future.

To understand the influence of risk variants on gene expression, several productive tools have been developed. For the chromatin states, ChIP-seq is an efficient genome-wide method to identify the transcription factor binding sites in open chromatin regions, including promoter, enhancer and other transcription active elements. Based on the principle of ChIP-seq, a series of targeted chromatin DNA sequencing technologies have been developed (*e.g.*, DNase-seq, MNase-seq, FAIRE-seq and ATAC-seq). For example, Forrest et al. revealed the function of non-coding GWAS risk variants using ATAC-seq data from neurons derived from SCZ patient induced pluripotent stem cells (iPSCs) [90]. Chip-related technology can help us to annotate and interpret the functionality of disease-associated non-coding variants. Data on DNA-protein binding generated by sequencing technologies requires validation using *in vitro* methods, including the electrophoretic mobility shift assays (EMSAs). However, the throughput of the EMSA-based experiments is limited. To improve the throughput of this *in vitro* validation, mass spectroscopy proteome-wide analysis of SNPs (PWAS) can be applied for screening genetic variants for differential transcription factor binding [91].

Risk variants located in the untranslated region (UTR) and intronic regions may also contribute to disease through post-transcriptional regulation, such as splicing, RNA stability, or non-coding regulation. High-throughput analysis of RNA isolated by cross-linking immunoprecipitation sequencing (CLIP-Seq) could be used to map protein-RNA binding site or RNA modification site *in vivo* [92], [93], [94]. This technique can reveal risk variants that affect gene expression at the post-transcriptional level. For example, Eric T. Wang used RNA-seq and CLIP-seq to reveal the transcriptome-wide regulation of pre-mRNA splicing and mRNA localization in myotonic dystrophy [95].

It is important to note that risk variants may not necessarily affect expression of the nearest gene. Disease risk variants may also affect expression of distal genes through long-range chromatin interactions [96], [97], [98]. The interaction of chromatin-specific regions can be explored by classic chromatin conformation capture (3C) techniques. This 3C-based technology involves cross-linking chromatin interaction sites, using genome DNA cleavage with a restriction enzyme and a ligation reaction to join cross-linked DNA fragments. Chromatin interactions at specific candidate loci could be further validated by polymerase chain reaction (PCR) [99]. For example, Panos Roussos et al. demonstrated physical interactions between the *CACNA1C* eQTL risk locus and distal regulatory elements using 3C techniques in prefrontal cortex [100].

The next step is to explore disease-associated phenotypes of genetic risk variants by establishing cellular models or animal models. For example, human iPSCs (hiPSCs) research detects molecular and cellular phenotypes (*e.g.*, migration, proliferation, and electrophysiology) together with the genetic background of specific patients. Moreover, the 3D culturing of pluripotent stem cells produces organoids, demonstrating their remarkable capacity for self-organization and differentiation. This approach can be used to study human brain specific features and the mechanism of neurodevelopment and neuropsychiatric disorders. For example, Marina Bershteyn et al. used human-derived cerebral organoids to model the cellular features of Miller-Dieker syndrome caused by 17p13.3 deletion [101]. While animal models differ from humans in terms of genetic background, they resemble the spectrum of human disease phenotypes, ranging from tissue and organ to behavior. Those two models, when combined with postmortem brain data, may unlock the mysteries of risk variant function and increase the probability of decoding the pathology of neuropsychiatric diseases.

## Future directions

In this review, we summarized the most representative brain banks and brain projects worldwide, supporting a multidimensional understanding of neuropsychiatric disorders from pathology, genetic, and gene expression perspectives. Brain banks and projects are establishing research resources and building coalitions to reduce the incidence and impact of neuropsychiatric disorders. Multidimensional data collected using brain bank resources facilitate the study of complex neuropsychiatric disorders, as brain banks are increasingly linked to important sources of clinical information. Different brain projects use brain bank samples to generate a wide spectrum of data types and serve as an important resource to promoting brain research. Developing advanced research methods and experimental validation of findings increases our capability of finding true causal signals of neuropsychiatric illnesses.

Postmortem brain samples have lent profound insight into genomic, transcriptomic and epigenomic studies, however brain disorder research faces many challenges. Various cell types from different brain regions form specific neural circuits that govern complex behaviors. Most brain studies include samples from different brain regions and use the bulk brain tissue as a whole, which obviously contains many cell types, such as neurons, astrocytes, microglia, and oligodendrocytes. Single-cell studies are increasingly needed to achieve higher resolution in detailed genomic insights. Some recent studies have been used single-cell methods to isolate specific cell types from healthy human brain tissue to characterize human brain development [102], [103]. Heterogeneity in medical treatment is one confounding factor that can affect gene expression profiles and some epigenetic marks. Almost every psychotic patient has a long history of drug therapy, but individuals without neuropsychiatric disorders may not, which may result in possible false-positive findings. Furthermore, integrating the drug history relies on obtaining hospital medical records or self-reporting, both of which can be unreliable. For example, patients may refuse to take prescribed medications, while others may not be able to accurately recall their medication history. Directed toxicology testing for each sample is the best solution but may not be practical due to the many types of antipsychotic drugs available and the high expense involved. Moreover, smoking and drinking history, state of death (*e.g.*, unexpected death, expired while asleep, unconsciousness, fever and hypoxia) are also confounding factors for postmortem gene expression and other studies [104], [105]. Consider this necessary information when collecting samples.

One vital but challenging aspect of brain collection is the use of fetal and infant brains. In most banks, donated brains come from aged individuals, appropriate for the research of neurodegenerative diseases. For neurodevelopmental diseases, such as autism, SCZ, and intellectual disability, however, fetal and infant brain samples are critical for investigating disease etiology. So far, only a few banks have prenatal samples, and their samples sizes are relatively small. Including fetuses with lethal defects and those with defects not affecting brain function, identified through prenatal genetic screening, could increase available resources. Another solution would be using iPSC-derived neurons or other brain cells to model the very early stages of brain development. Combining these strategies, we can characterize the temporal regulatory landscape of brain development and genomic aberrations related to psychiatric illnesses.

Recently, it has been suggested that all postmortem brain studies are underpowered to correct for genetic and phenotypic heterogeneity [106]. This begs the question, how can these studies derive from the brain banks with limited sample sizes achieve enough statistical power? One solution is in more accurately defining disease-related phenotyping and levels of disease taxonomy. For example, in BIP, only about 30% of patients respond to lithium [107], [108], and a portion of patients have DLPFC or hippocampal volume abnormalities [109], [110], [111], [112]. Classification of these disease subtypes improves the understanding of disease phenotype. Availability of shared data is another big issue often limiting the power needed for research into neuropsychiatric disorders. With more and more data generated and released, an open public and user-interactive data center is needed to collect and to manage all the repositories. Our group established the Brain EXPression Database (BrainEXP, http://www.brainexp.org/) focusing on brain gene expression patterns in various regions, by sex and age [113]. This database currently includes 4567 brain samples of 2863 normal individuals and will integrate approximately the same number of patient samples in the near future. These combined efforts hold the promise of powering brain studies adequately.

In conclusion, given the expanding framework of brain bank and brain project networks, we can improve exploration into the molecular regulatory mechanisms of neuropsychiatric disorders and facilitate research toward new avenues of treatment.

## Competing interests

The authors have declared no competing interests.

## References

[1] Hyman S.E. (2012). Revolution stalled. Sci Transl Med.

[2] Cardno A.G., Gottesman I.I. (2000). Twin studies of schizophrenia: from bow-and-arrow concordances to star wars Mx and functional genomics. Am J Med Genet.

[3] Sullivan P.F., Kendler K.S., Neale M.C. (2003). Schizophrenia as a complex trait: evidence from a meta-analysis of twin studies. Arch Gen Psychiatry.

[4] Sullivan P.F. (2010). The psychiatric GWAS consortium: big science comes to psychiatry. Neuron.

[5] Stahl E.A., Breen G., Forstner A.J., McQuillin A., Ripke S., Trubetskoy V. (2019). Genome-wide association study identifies 30 loci associated with bipolar disorder. Nat Genet.

[6] Wray N.R., Ripke S., Mattheisen M., Trzaskowski M., Byrne E.M., Abdellaoui A. (2018). Genome-wide association analyses identify 44 risk variants and refine the genetic architecture of major depression. Nat Genet.

[7] CONVERGE Consortium (2015). Sparse whole genome sequencing identifies two loci for major depressive disorder. Nature.

[8] Lappalainen T., Sammeth M., Friedlander M.R., t Hoen P.A., Monlong J., Rivas M.A. (2013). Transcriptome and genome sequencing uncovers functional variation in humans. Nature.

[9] Zhang D., Cheng L., Badner J.A., Chen C., Chen Q., Luo W. (2010). Genetic control of individual differences in gene-specific methylation in human brain. Am J Hum Genet.

[10] Waszak S.M., Delaneau O., Gschwind A.R., Kilpinen H., Raghav S.K., Witwicki R.M. (2015). Population variation and genetic control of modular chromatin architecture in humans. Cell.

[11] Grubert F., Zaugg J.B., Kasowski M., Ursu O., Spacek D.V., Martin A.R. (2015). Genetic control of chromatin states in humans involves local and distal chromosomal interactions. Cell.

[12] Li M., Wu D.D., Yao Y.G., Huo Y.X., Liu J.W., Su B. (2016). Recent positive selection drives the expansion of a schizophrenia risk nonsynonymous variant at *SLC39A8* in Europeans. Schizophr Bull.

[13] Ongen H., Brown A.A., Delaneau O., Panousis N.I., Nica A.C., Consortium GT (2017). Estimating the causal tissues for complex traits and diseases. Nat Genet.

[14] Murthy M.N., Blauwendraat C., UK Brain Expression Consortium, Guelfi S., The International Parkinson Disease Genomics Consortium, Hardy J. (2017). Increased brain expression of *GPNMB* is associated with genome wide significant risk for Parkinson’s disease on chromosome 7p15.3. Neurogenetics.

[15] Kretzschmar H. (2009). Brain banking: opportunities, challenges and meaning for the future. Nat Rev Neurosci.

[16] Bell J.E., Alafuzoff I., Al-Sarraj S., Arzberger T., Bogdanovic N., Budka H. (2008). Management of a twenty-first century brain bank: experience in the BrainNet Europe consortium. Acta Neuropathol.

[17] Nichols L., Freund M., Ng C., Kau A., Parisi M., Taylor A. (2014). The National Institutes of Health Neurobiobank: a federated national network of human brain and tissue repositories. Biol Psychiatry.

[18] Palmer-Aronsten B., Sheedy D., McCrossin T., Kril J. (2016). An international survey of brain banking operation and characterization practices. Biopreserv Biobank.

[19] Stan A.D., Ghose S., Gao X.M., Roberts R.C., Lewis-Amezcua K., Hatanpaa K.J. (2006). Human postmortem tissue: what quality markers matter?. Brain Res.

[20] White K., Yang P., Li L., Farshori A., Medina A.E., Zielke H.R. (2018). Effect of postmortem interval and years in storage on RNA quality of tissue at a repository of the NIH NeuroBioBank. Biopreserv Biobank.

[21] Schmitt A., Parlapani E., Bauer M., Heinsen H., Falkai P. (2008). Is brain banking of psychiatric cases valuable for neurobiological research?. Clinics (Sao Paulo).

[22] Yan X.X., Ma C., Bao A.M., Wang X.M., Gai W.P. (2015). Brain banking as a cornerstone of neuroscience in China. Lancet Neurol.

[23] Zhang H., Chen K., Wang N., Zhang D., Yang Q., Zhang Q. (2018). Analysis of brain donors' demographic and medical characteristics to facilitate the construction of a human brain bank in China. J Alzheimers Dis.

[24] Pipes L., Li S., Bozinoski M., Palermo R., Peng X., Blood P. (2013). The non-human primate reference transcriptome resource (NHPRTR) for comparative functional genomics. Nucleic Acids Res.

[25] Doan R.N., Bae B.I., Cubelos B., Chang C., Hossain A.A., Al-Saad S. (2016). Mutations in human accelerated regions disrupt cognition and social behavior. Cell.

[26] Samarasekera N., Al-Shahi Salman R., Huitinga I., Klioueva N., McLean C.A., Kretzschmar H. (2013). Brain banking for neurological disorders. Lancet Neurol.

[27] Miller J.A., Ding S.L., Sunkin S.M., Smith K.A., Ng L., Szafer A. (2014). Transcriptional landscape of the prenatal human brain. Nature.

[28] Hawrylycz M.J., Lein E.S., Guillozet-Bongaarts A.L., Shen E.H., Ng L., Miller J.A. (2012). An anatomically comprehensive atlas of the adult human brain transcriptome. Nature.

[29] Ramasamy A., Trabzuni D., Guelfi S., Varghese V., Smith C., Walker R. (2014). Genetic variability in the regulation of gene expression in ten regions of the human brain. Nat Neurosci.

[30] GTEx Consortium (2013). The Genotype-Tissue Expression (GTEx) project. Nat Genet.

[31] Fromer M., Roussos P., Sieberts S.K., Johnson J.S., Kavanagh D.H., Perumal T.M. (2016). Gene expression elucidates functional impact of polygenic risk for schizophrenia. Nat Neurosci.

[32] BrainSeq: A Human Brain Genomics Consortium (2015). BrainSeq: neurogenomics to drive novel target discovery for neuropsychiatric disorders. Neuron.

[33] De Jager P.L., Srivastava G., Lunnon K., Burgess J., Schalkwyk L.C., Yu L. (2014). Alzheimer's disease: early alterations in brain DNA methylation at *ANK1*, *BIN1*, *RHBDF2* and other loci. Nat Neurosci.

[34] PsychENCODE Consortium, Akbarian S., Liu C., Knowles J.A., Vaccarino F.M., Farnham P.J. (2015). The PsychENCODE project. Nat Neurosci.

[35] Colantuoni C., Lipska B.K., Ye T., Hyde T.M., Tao R., Leek J.T. (2011). Temporal dynamics and genetic control of transcription in the human prefrontal cortex. Nature.

[36] Bennett D.A., Schneider J.A., Arvanitakis Z., Wilson R.S. (2012). Overview and findings from the religious orders study. Curr Alzheimer Res.

[37] Bennett D.A., Schneider J.A., Buchman A.S., Barnes L.L., Boyle P.A., Wilson R.S. (2012). Overview and findings from the rush memory and aging project. Curr Alzheimer Res.

[38] Li M., Santpere G., Imamura Kawasawa Y., Evgrafov O.V., Gulden F.O., Pochareddy S. (2018). Integrative functional genomic analysis of human brain development and neuropsychiatric risks. Science.

[39] Wang D., Liu S., Warrell J., Won H., Shi X., Navarro F.C.P. (2018). Comprehensive functional genomic resource and integrative model for the human brain. Science.

[40] Rajarajan P., Borrman T., Liao W., Schrode N., Flaherty E., Casino C. (2018). Neuron-specific signatures in the chromosomal connectome associated with schizophrenia risk. Science.

[41] Jaffe A.E., Straub R.E., Shin J.H., Tao R., Gao Y., Collado-Torres L. (2018). Developmental and genetic regulation of the human cortex transcriptome illuminate schizophrenia pathogenesis. Nat Neurosci.

[42] Jaffe A.E., Gao Y., Deep-Soboslay A., Tao R., Hyde T.M., Weinberger D.R. (2016). Mapping DNA methylation across development, genotype and schizophrenia in the human frontal cortex. Nat Neurosci.

[43] Zhu Y., Sousa A.M.M., Gao T., Skarica M., Li M., Santpere G. (2018). Spatiotemporal transcriptomic divergence across human and macaque brain development.. Science.

[44] Gandal M.J., Zhang P., Hadjimichael E., Walker R.L., Chen C., Liu S. (2018). Transcriptome-wide isoform-level dysregulation in ASD, schizophrenia, and bipolar disorder. Science.

[45] Meng Q., Wang K., Brunetti T., Xia Y., Jiao C., Dai R. (2018). The *DGCR5* long noncoding RNA may regulate expression of several schizophrenia-related genes. Sci Transl Med.

[46] Chen C., Meng Q., Xia Y., Ding C., Wang L., Dai R. (2018:). The transcription factor *POU3F2* regulates a gene coexpression network in brain tissue from patients with psychiatric disorders. Sci Transl Med.

[47] Locascio J.J., Atri A. (2011). An overview of longitudinal data analysis methods for neurological research. Dement Geriatr Cogn Dis Extra.

[48] Conesa A., Madrigal P., Tarazona S., Gomez-Cabrero D., Cervera A., McPherson A. (2016). A survey of best practices for RNA-seq data analysis. Genome Biol.

[49] Teng M., Love M.I., Davis C.A., Djebali S., Dobin A., Graveley B.R. (2016). A benchmark for RNA-seq quantification pipelines. Genome Biol.

[50] Trapnell C., Williams B.A., Pertea G., Mortazavi A., Kwan G., van Baren M.J. (2010). Transcript assembly and quantification by RNA-seq reveals unannotated transcripts and isoform switching during cell differentiation. Nat Biotechnol.

[51] Roberts A., Pachter L. (2013). Streaming fragment assignment for real-time analysis of sequencing experiments. Nat Methods.

[52] Montgomery S.B., Sammeth M., Gutierrez-Arcelus M., Lach R.P., Ingle C., Nisbett J. (2010). Transcriptome genetics using second generation sequencing in a Caucasian population. Nature.

[53] Bray N.L., Pimentel H., Melsted P., Pachter L. (2016). Near-optimal probabilistic RNA-seq quantification. Nat Biotechnol.

[54] Li B., Dewey C.N. (2011). RSEM: accurate transcript quantification from RNA-Seq data with or without a reference genome. BMC Bioinformatics.

[55] Patro R., Mount S.M., Kingsford C. (2014). Sailfish enables alignment-free isoform quantification from RNA-seq reads using lightweight algorithms. Nat Biotechnol.

[56] Patro R., Duggal G., Love M.I., Irizarry R.A., Kingsford C. (2017). Salmon provides fast and bias-aware quantification of transcript expression. Nat Methods.

[57] GTEx Consortium (2017). Genetic effects on gene expression across human tissues. Nature.

[58] Gandal M.J., Haney J.R., Parikshak N.N., Leppa V., Ramaswami G., Hartl C. (2018). Shared molecular neuropathology across major psychiatric disorders parallels polygenic overlap. Science.

[59] Oldham M.C., Langfelder P., Horvath S. (2012). Network methods for describing sample relationships in genomic datasets: application to Huntington's disease. BMC Syst Biol.

[60] Tsoucas D., Dong R., Chen H., Zhu Q., Guo G., Yuan G.C. (2019). Accurate estimation of cell-type composition from gene expression data. Nat Commun.

[61] Stegle O., Parts L., Piipari M., Winn J., Durbin R. (2012). Using probabilistic estimation of expression residuals (PEER) to obtain increased power and interpretability of gene expression analyses. Nat Protoc.

[62] Leek J.T., Johnson W.E., Parker H.S., Jaffe A.E., Storey J.D. (2012). The sva package for removing batch effects and other unwanted variation in high-throughput experiments. Bioinformatics.

[63] Fusi N., Stegle O., Lawrence N.D. (2012). Joint modelling of confounding factors and prominent genetic regulators provides increased accuracy in genetical genomics studies. PLoS Comput Biol.

[64] Gaffney D.J., Veyrieras J.B., Degner J.F., Pique-Regi R., Pai A.A., Crawford G.E. (2012). Dissecting the regulatory architecture of gene expression QTLs. Genome Biol.

[65] van de Geijn B., McVicker G., Gilad Y., Pritchard J.K. (2015). WASP: allele-specific software for robust molecular quantitative trait locus discovery. Nat Methods.

[66] Zhu Z., Zhang F., Hu H., Bakshi A., Robinson M.R., Powell J.E. (2016). Integration of summary data from GWAS and eQTL studies predicts complex trait gene targets. Nat Genet.

[67] Ward M.C., Gilad Y. (2017). Human genomics: cracking the regulatory code. Nature.

[68] Pavlides J.M., Zhu Z., Gratten J., McRae A.F., Wray N.R., Yang J. (2016). Predicting gene targets from integrative analyses of summary data from GWAS and eQTL studies for 28 human complex traits. Genome Med.

[69] Barbeira A., Dickinson S.P., Bonazzola R., Zheng J., Wheeler H.E., Torres J.M. (2018). Exploring the phenotypic consequences of tissue specific gene expression variation inferred from GWAS summary statistics. Nat Commun.

[70] Gamazon E.R., Wheeler H.E., Shah K.P., Mozaffari S.V., Aquino-Michaels K., Carroll R.J. (2015). A gene-based association method for mapping traits using reference transcriptome data. Nat Genet.

[71] Gusev A., Ko A., Shi H., Bhatia G., Chung W., Penninx B.W. (2016). Integrative approaches for large-scale transcriptome-wide association studies. Nat Genet.

[72] Giambartolomei C., Vukcevic D., Schadt E.E., Franke L., Hingorani A.D., Wallace C. (2014). Bayesian test for colocalisation between pairs of genetic association studies using summary statistics. PLoS Genet.

[73] Giambartolomei C., Liu J.Z., Zhang W., Hauberg M., Shi H., Boocock J. (2018). A Bayesian framework for multiple trait colocalization from summary association statistics. Bioinformatics.

[74] Foley CN, Staley JR, Breen PG, Sun BB, Kirk PDW, Burgess S, et al. A fast and efficient colocalization algorithm for identifying shared genetic risk factors across multiple traits. bioRxiv 2019;592238. 10.1038/s41467-020-20885-8 PMC7858636 33536417

[75] He X., Fuller C.K., Song Y., Meng Q., Zhang B., Yang X. (2013). Sherlock: detecting gene-disease associations by matching patterns of expression QTL and GWAS. Am J Hum Genet.

[76] Lee D., Williamson V.S., Bigdeli T.B., Riley B.P., Fanous A.H., Vladimirov V.I. (2015). JEPEG: a summary statistics based tool for gene-level joint testing of functional variants. Bioinformatics.

[77] Hormozdiari F., Kostem E., Kang E.Y., Pasaniuc B., Eskin E. (2014). Identifying causal variants at loci with multiple signals of association. Genetics.

[78] Hormozdiari F., van de Bunt M., Segre A.V., Li X., Joo J.W.J., Bilow M. (2016). Colocalization of GWAS and eQTL signals detects target genes. Am J Hum Genet.

[79] Yang F., Wang J., Consortium GT, Pierce B.L., Chen L.S. (2017). Identifying cis-mediators for trans-eQTLs across many human tissues using genomic mediation analysis. Genome Res.

[80] Benner C., Spencer C.C., Havulinna A.S., Salomaa V., Ripatti S., Pirinen M. (2016). FINEMAP: efficient variable selection using summary data from genome-wide association studies. Bioinformatics.

[81] Gamazon E.R., Segre A.V., van de Bunt M., Wen X., Xi H.S., Hormozdiari F. (2018). Using an atlas of gene regulation across 44 human tissues to inform complex disease- and trait-associated variation. Nat Genet.

[82] Bulik-Sullivan B.K., Loh P.R., Finucane H.K., Ripke S., Yang J., Schizophrenia Working Group of the Psychiatric Genomics C (2015). LD Score regression distinguishes confounding from polygenicity in genome-wide association studies. Nat Genet.

[83] Aten J.E., Fuller T.F., Lusis A.J., Horvath S. (2008). Using genetic markers to orient the edges in quantitative trait networks: the NEO software. BMC Syst Biol.

[84] Zhu Z., Zheng Z., Zhang F., Wu Y., Trzaskowski M., Maier R. (2018). Causal associations between risk factors and common diseases inferred from GWAS summary data. Nat Commun.

[85] Battle A., Mostafavi S., Zhu X., Potash J.B., Weissman M.M., McCormick C. (2014). Characterizing the genetic basis of transcriptome diversity through RNA-sequencing of 922 individuals. Genome Res.

[86] Wen X., Pique-Regi R., Luca F. (2017). Integrating molecular QTL data into genome-wide genetic association analysis: Probabilistic assessment of enrichment and colocalization. PLoS Genet.

[87] Li X., Kim Y., Tsang E.K., Davis J.R., Damani F.N., Chiang C. (2017). The impact of rare variation on gene expression across tissues. Nature.

[88] Diao Y., Fang R., Li B., Meng Z., Yu J., Qiu Y. (2017). A tiling-deletion-based genetic screen for cis-regulatory element identification in mammalian cells. Nat Methods.

[89] Zhang X.H., Tee L.Y., Wang X.G., Huang Q.S., Yang S.H. (2015). Off-target effects in CRISPR/Cas9-mediated genome engineering. Mol Ther Nucleic Acids.

[90] Forrest M.P., Zhang H., Moy W., McGowan H., Leites C., Dionisio L.E. (2017). Open chromatin profiling in hiPSC-derived neurons prioritizes functional noncoding psychiatric risk variants and highlights neurodevelopmental loci. Cell Stem Cell.

[91] Butter F., Davison L., Viturawong T., Scheibe M., Vermeulen M., Todd J.A. (2012). Proteome-wide analysis of disease-associated SNPs that show allele-specific transcription factor binding. PLoS Genet.

[92] Darnell R.B. (2010). HITS-CLIP: panoramic views of protein-RNA regulation in living cells. Wiley Interdiscip Rev RNA.

[93] Licatalosi D.D., Mele A., Fak J.J., Ule J., Kayikci M., Chi S.W. (2008). HITS-CLIP yields genome-wide insights into brain alternative RNA processing. Nature.

[94] Ke S., Alemu E.A., Mertens C., Gantman E.C., Fak J.J., Mele A. (2015). A majority of m^6^A residues are in the last exons, allowing the potential for 3' UTR regulation. Genes Dev.

[95] Wang E.T., Cody N.A., Jog S., Biancolella M., Wang T.T., Treacy D.J. (2012). Transcriptome-wide regulation of pre-mRNA splicing and mRNA localization by muscleblind proteins. Cell.

[96] French J.D., Ghoussaini M., Edwards S.L., Meyer K.B., Michailidou K., Ahmed S. (2013). Functional variants at the 11q13 risk locus for breast cancer regulate *cyclin D1* expression through long-range enhancers. Am J Hum Genet.

[97] Praetorius C., Grill C., Stacey S.N., Metcalf A.M., Gorkin D.U., Robinson K.C. (2013). A polymorphism in *IRF4* affects human pigmentation through a tyrosinase-dependent MITF/TFAP2A pathway. Cell.

[98] Claussnitzer M., Dankel S.N., Kim K.H., Quon G., Meuleman W., Haugen C. (2015). *FTO* obesity variant circuitry and adipocyte browning in humans. N Engl J Med.

[99] Rhie S.K., Coetzee S.G., Noushmehr H., Yan C., Kim J.M., Haiman C.A. (2013). Comprehensive functional annotation of seventy-one breast cancer risk Loci. PLoS One.

[100] Roussos P., Mitchell A.C., Voloudakis G., Fullard J.F., Pothula V.M., Tsang J. (2014). A role for noncoding variation in schizophrenia. Cell Rep.

[101] Bershteyn M., Nowakowski T.J., Pollen A.A., Di Lullo E., Nene A., Wynshaw-Boris A. (2017). Human iPSC-derived cerebral organoids model cellular features of lissencephaly and reveal prolonged mitosis of outer radial glia. Cell Stem Cell.

[102] Fan X., Dong J., Zhong S., Wei Y., Wu Q., Yan L. (2018). Spatial transcriptomic survey of human embryonic cerebral cortex by single-cell RNA-seq analysis. Cell Res.

[103] Lake B.B., Ai R., Kaeser G.E., Salathia N.S., Yung Y.C., Liu R. (2016). Neuronal subtypes and diversity revealed by single-nucleus RNA sequencing of the human brain. Science.

[104] Aggarwal N.T., Bienias J.L., Bennett D.A., Wilson R.S., Morris M.C., Schneider J.A. (2006). The relation of cigarette smoking to incident Alzheimer's disease in a biracial urban community population. Neuroepidemiology.

[105] Wilson R.S., Bienias J.L., Mendes de Leon C.F., Evans D.A., Bennett D.A. (2003). Negative affect and mortality in older persons. Am J Epidemiol.

[106] Sabunciyan S. (2019). Gene expression profiles associated with brain aging are altered in schizophrenia. Sci Rep.

[107] Viguera A.C., Tondo L., Baldessarini R.J. (2000). Sex differences in response to lithium treatment. Am J Psychiatry.

[108] Tohen M., Greil W., Calabrese J.R., Sachs G.S., Yatham L.N., Oerlinghausen B.M. (2005). Olanzapine versus lithium in the maintenance treatment of bipolar disorder: a 12-month, randomized, double-blind, controlled clinical trial. Am J Psychiatry.

[109] Rajkowska G., Halaris A., Selemon L.D. (2001). Reductions in neuronal and glial density characterize the dorsolateral prefrontal cortex in bipolar disorder. Biol Psychiatry.

[110] Bertolino A., Frye M., Callicott J.H., Mattay V.S., Rakow R., Shelton-Repella J. (2003). Neuronal pathology in the hippocampal area of patients with bipolar disorder: a study with proton magnetic resonance spectroscopic imaging. Biol Psychiatry.

[111] Bouras C., Kovari E., Hof P.R., Riederer B.M., Giannakopoulos P. (2001). Anterior cingulate cortex pathology in schizophrenia and bipolar disorder. Acta Neuropathol.

[112] Rajkowska G. (2000). Postmortem studies in mood disorders indicate altered numbers of neurons and glial cells. Biol Psychiatry.

[113] Jiao C., Yan P., Xia C., Shen Z., Tan Z., Tan Y. (2019). BrainEXP: a database featuring with spatiotemporal expression variations and co-expression organizations in human brains. Bioinformatics.

